# Bryophyte gas‐exchange dynamics along varying hydration status reveal a significant carbonyl sulphide (COS) sink in the dark and COS source in the light

**DOI:** 10.1111/nph.14584

**Published:** 2017-05-03

**Authors:** Teresa E. Gimeno, Jérôme Ogée, Jessica Royles, Yves Gibon, Jason B. West, Régis Burlett, Sam P. Jones, Joana Sauze, Steven Wohl, Camille Benard, Bernard Genty, Lisa Wingate

**Affiliations:** ^1^ ISPA Bordeaux Science Agro INRA Villenave d'Ornon 33140 France; ^2^ Department Plant Sciences University of Cambridge Cambridge CB2 3EA UK; ^3^ UMR BFP 1332 Plateforme Métabolome du Centre de Génomique Fonctionnelle Bordeaux PHENOME INRA University of Bordeaux Villenave d'Ornon 33140 France; ^4^ Department of Ecosystem Science & Management Texas A&M University College Station TX 77845 USA; ^5^ UMR BIOGECO INRA University of Bordeaux Talence 33450 France; ^6^ CNRS/CEA/Aix‐Marseille University UMR 7265 BVME Saint‐Paul‐lez‐Durance France

**Keywords:** carbohydrates, desiccation, liverwort, *Marchantia polymorpha*, moss, protein, respiration, *Scleropodium purum*

## Abstract

- Carbonyl sulphide (COS) is a potential tracer of gross primary productivity (GPP), assuming a unidirectional COS flux into the vegetation that scales with GPP. However, carbonic anhydrase (CA), the enzyme that hydrolyses COS, is expected to be light independent, and thus plants without stomata should continue to take up COS in the dark.
- We measured net CO_2_ (*A*^C^) and COS (*A*^S^) uptake rates from two astomatous bryophytes at different relative water contents (RWCs), COS concentrations, temperatures and light intensities.
- We found large *A*^S^ in the dark, indicating that CA activity continues without photosynthesis. More surprisingly, we found a nonzero COS compensation point in light and dark conditions, indicating a temperature‐driven COS source with a *Q*_10_ (fractional change for a 10°C temperature increase) of 3.7. This resulted in greater *A*^S^ in the dark than in the light at similar RWC. The processes underlying such COS emissions remain unknown.
- Our results suggest that ecosystems dominated by bryophytes might be strong atmospheric sinks of COS at night and weaker sinks or even sources of COS during daytime. Biotic COS production in bryophytes could result from symbiotic fungal and bacterial partners that could also be found on vascular plants.

## Abstract 2

See also the Commentary on this article by Wohlfahrt, **215**: 923–925.

## Introduction

Carbonyl sulphide (COS) is the most abundant sulphur‐containing gas in the troposphere and has the potential to serve as a proxy for estimating gross primary productivity (GPP, Sandoval‐Soto *et al*., 2005; Montzka *et al*., 2007; Campbell *et al*., 2008). The foundation for using COS as a GPP tracer is built on the assumption that terrestrial uptake is dominated by, and proportional to, plant photosynthetic activity. This is because COS is taken up by plants following a similar pathway to that of CO_2_. COS diffuses into the vegetation through the stomatal pores and is hydrolysed by the enzyme carbonic anhydrase (CA) in the mesophyll cells (Protoschill‐Krebs *et al*., 1996). However, in contrast to CO_2_ hydration by CA, COS hydrolysis by CA is irreversible (Notni *et al*., 2007) and no other leaf‐level processes have been identified in the production of COS (Bloem *et al*., 2015). Thus, COS uptake (*A* ^S^) is assumed to be unidirectional and not the net result of two opposed fluxes (photosynthesis and respiration in the case of CO_2_). This assumption is key for the calculation of GPP from COS fluxes, together with an estimate of the ratio between CO_2_ and COS uptake, the so‐called ‘leaf relative uptake’ (LRU, Campbell *et al*., 2008). Relying on LRU for estimating GPP requires some important assumptions. Principally, the LRU approach assumes that the consumption of CO_2_ and COS diffusing into the leaf is linked to downstream light‐dependent reactions. However, CA activity is expected to be light independent (Protoschill‐Krebs *et al*., 1996). Thus, as long as stomata remain open, a sink for COS should be maintained in the dark when CO_2_ uptake (*A* ^C^) ceases and *A* ^C^ becomes dominated by leaf respiration. Indeed an uncoupling of *A* ^C^ and *A* ^S^ (i.e. more variable and usually larger LRU values) has been reported at low light intensities in the lab (Stimler *et al*., 2011) and in the field at night (Berkelhammer *et al*., 2014; Commane *et al*., 2015). Furthermore, the utility of COS as a tracer of GPP depends heavily on the assumption that the flux of COS between the atmosphere and the leaf is one‐way and driven by CA activity alone. This assumption has been validated at the leaf level for certain species and environmental conditions (Stimler *et al*., 2010; Sandoval‐Soto *et al*., 2012). However, recent field studies have shown that COS emissions from wheat leaves may occur during senescence and from deciduous forests during periods of high temperature and drought (Maseyk *et al*., 2014; Commane *et al*., 2015). Thus it is not entirely clear whether the unidirectional hypothesis holds in plants exposed to stress and whether COS emissions are masked to some extent by the stomata of vascular plants. Currently, the mechanisms underlying these COS emissions remain unclear.

Plants without stomata such as bryophytes are a potentially useful model that could provide important insights into the dynamics of *A* ^S^ under varying environmental conditions. These dynamics would otherwise be difficult to detect in the presence of stomata that actively impose diffusional limitations. In astomatous plants, the dynamics of leaf COS fluxes should be more closely related to changes in the enzymatic activity and substrate availability. Disentangling diffusional and enzymatic processes for plant COS fluxes may be especially critical since stomata have been shown to open in response to an increase in the atmospheric COS mixing ratio (Stimler *et al*., 2010, 2012), creating a potential feedback on leaf COS and CO_2_ uptake. Although some bryophytes have tiny pores that facilitate gas exchange, they lack stomatal regulation (Proctor *et al*., 2007) and seem therefore better suited for assessment of changes in leaf COS fluxes in response to changing environmental conditions.

Bryophytes lack active control of transpiration so they rapidly equilibrate with prevailing environmental conditions (Proctor *et al*., 2007). Desiccation tolerance in these organisms involves a number of biochemical mechanisms such as the accumulation of nonstructural carbohydrates and other compatible solutes or the up‐ or downregulation of gene expression and protein synthesis (Oliver *et al*., 2005). To reduce the rate of water loss, bryophytes also deploy morphological adaptations and preserve a thin layer of capillary water on their leafy shoots (Marschall & Proctor, 2004). Capillary water slows tissue desiccation, but it is a barrier to CO_2_ diffusion. As a consequence, bryophyte *A* ^C^ commonly displays a three‐phase response to tissue dehydration. Initially, as diffusion‐resistance through capillary water decreases with evaporation, *A* ^C^ increases until a plateau in *A* ^C^ is reached indicating the optimal hydration status for photosynthetic activity. As evaporation continues, *A* ^C^ decreases as cells dehydrate and photosynthesis becomes metabolically impaired (Dilks & Proctor, 1979; Royles *et al*., 2013). This layer of capillary water should pose a resistance not only to CO_2_, but also to COS, and thus we suggest that a similar optimum‐like response of *A* ^S^ to desiccation should be observed in the light. Alternatively, in bryophytes with carbon concentration mechanisms similar to those of algae and cyanobacteria (Smith & Griffiths, 2000), external CA activity could potentially counterbalance the initial expected increase in *A* ^S^ with desiccation (Rech *et al*., 2008). In the dark, *A* ^C^ flux is negative as respiration dominates and the magnitude of the respiratory flux decreases progressively with desiccation. However, in the absence of stomata and because CA is expected to be light‐independent (Gries *et al*., 1994; Protoschill‐Krebs *et al*., 1996), we hypothesise that *A* ^S^ should continue at similar rates to those observed in the light.

Bryophytes should also be a good model to test the assumption that COS emission does not occur during the day/night cycle or in plants exposed to water or heat stress. Testing this hypothesis on vascular plants would be challenging, as *A* ^S^ would be strongly limited by stomatal closure. The only previous study estimating the COS compensation point (Γ^S^, the COS concentration at which *A* ^S^ is zero) on nonvascular photoautotrophic organisms (lichens, Kuhn & Kesselmeier, 2000) suggested that Γ^S^ could be greater than zero. A positive Γ^S^ would imply that *A* ^S^ is the net result of simultaneous COS uptake and emission. As far as we are aware no studies looking into the relationship between *A* ^S^ and the COS mixing ratio (*C* ^S^) have been conducted on astomatous plants to date.

Here, we challenge our current understanding of COS uptake by terrestrial plants using astomatous bryophytes as model organisms. Our aims were to provide a first estimate of the COS sink strength in bryophytes and to test some of the assumptions that underlie the proposed relationship between GPP and COS uptake. Specifically we hypothesised that (1) in astomatous bryophytes, COS uptake varies with tissue hydration analogously to CO_2_ uptake in the light, (2) COS uptake dynamics during desiccation would be similar in the light and in the dark and (3) the COS compensation point would be zero. To test these hypotheses we ran a series of experiments under controlled conditions to characterise the response of *A* ^S^ and *A* ^C^ to desiccation, COS concentration and increasing light intensity and temperature, in two bryophyte species with contrasting life forms and evolutionary origin.

## Materials and Methods

### Study species and sampling protocol

We chose two bryophytes with contrasting evolutionary origins and life forms, representative of temperate regions. The mat‐forming liverwort *Marchantia polymorpha* L. has gametophytes with a complex thallus structure and occasional static pores to improve ventilation (Meyer *et al*., 2008). The loosely packed weft‐moss *Scleropodium purum* (Hedw.) Limpr. is a desiccation‐tolerant slow‐growing moss with feather‐like shoots and poorly developed rhizoids (Arroniz‐Crespo *et al*., 2008). Given their abundance and widespread distribution, both species have been the subjects of an ample body of literature. *M. polymorpha* has served as a model bryophyte for characterising plant physiology, metabolism and genetics (Bowman, 2016) while *S. purum* physiology and distribution have been widely studied in response to nutrient availability and heavy metal contamination (Arroniz‐Crespo *et al*., 2008).

Mats of moss and liverwort were collected locally from naturally growing populations at the INRA campuses of ‘La Ferrade’ (Villenave d'Ornon, France) and ‘Pierroton’ (Cestas, France). Mats of *M. polymorpha* were occasionally intermingled with *Lunularia cruciata*, a liverwort with similar gametophytes to those of *M. polymorpha* in the absence of spore‐bearing cups. The mats were collected 1–5 d before the experiments and maintained in ambient external light with regular watering. On the day before each experiment, green tissue was separated and rinsed with deionised (DI) water. Individual samples of 2.5–4 g (fresh mass) of green tissue were placed onto pierced aluminium circular trays (6.5 cm diameter). To fully rehydrate the tissue, sample trays were sprayed with DI water, placed onto moist paper and kept refrigerated in closed glass jars for 12–24 h. Before the start of the experiment, the jars were acclimated to room temperature for 1 h. After blotting excess water from the trays, all trays were weighed to the nearest 0.1 mg and placed into gas‐exchange chambers, a few minutes before the experiment. All trays were reweighed at the end of each experiment.

### Gas‐exchange measurements

Experiments were carried out at the facility for online trace gas and stable isotope analyses at INRA‐Bordeaux (France). The system comprised a set of gas analysers that measured CO_2_, COS and H_2_O mixing ratios of the inlet and outlet airstreams from seven multiplexed gas‐exchange chambers. Each chamber consisted of a 0.5 l glass jar and a glass top fitted with two stainless steel Swagelok^®^ (Swagelok, Solon, OH, USA) connectors attached to 0.25 inch (3.175 mm) Teflon™ (Teflon, Chemours, Wilmington, DE, USA) inlet and outlet lines. The gas‐exchange chambers were housed in a climatically controlled chamber that regulated air temperature, relative humidity and light intensity, outside the chambers (MD1400; Snijders, Tillburg, the Netherlands). Inside each chamber, temperature was monitored continuously with self‐contained thermocouple data‐loggers (Hygrochron Temperature & Humidity iButton, DS1923; Embedded Data Systems, Lawrenceburg, KY, USA), placed on the aluminium trays in direct contact with the samples but not completely covered by them. Air pressure inside the chambers was established with a pressure transducer (BMP180; Bosch GmbH, Gerlingen, Germany), during preliminary experiments with the exact same flow of air and measuring sequence.

The airflow into each chamber was set to 250 ml min^−1^ on a dry air basis using individual mass‐flow controllers (MFC, EL‐Flow^®^ Select; Bronkhorst, Ruurlo, the Netherlands). CO_2_ and COS mixing ratios (*C* ^C^ and *C* ^S^, respectively) of the inlet air were adjusted by mixing synthetic CO_2_‐ and COS‐free dry air from a compressor (FM2 Atlas Copto, Nacka, Sweden), coupled to a chemical scrub column (Ecodry K‐MT6; Parker Hannifin, Cleveland, OH, USA), with two cylinders of commercial gas mixtures (pure CO_2_ and 500 nmol mol^−1^ COS). Inlet and outlet *C* ^C^ and *C* ^S^ were pre‐dried with a Nafion™ dryer (MD‐070‐24‐S‐2; Perma Pure LLC, Lakewood, NJ, USA) before being measured with a mid‐infrared quantum cascade laser spectrometer (QCLS; Aerodyne Research Inc., Billerica, MA, USA). Flow through the instrument was maintained with a TriScroll 600 pump (Agilent Technologies, Santa Clara, CA, USA) connected to the QCLS via a vacuum line. Instrument drift was corrected with frequent (every 14 min) background calibrations (with dry N_2_) in all runs. In most runs (75%) a two‐point standard calibration was also implemented using dry N_2_ (zero) and compressed dry air with a COS concentration of 524.8 ± 2.2 pmol mol^−1^ from an Aculife^®^ (Air Liquide USA, Houston, TX, USA)‐treated cylinder that was prepared and calibrated for COS by the NOAA Global Monitoring Division. The 14 min frequency was based on instrument stability estimated from an Allan variance calculated from a 24 h continuous measurement on tank air that indicated a standard deviation at 10 s averaging of 2.1 pmol mol^−1^ for COS, a deviation from pure white noise after > 400 s and a SD <1 pmol mol^−1^ after 900 s integration time (Supporting Information Fig. S1).

The QCLS alternately measured inlet and outlet *C* ^C^ and *C* ^S^ over 120 s and only the mean of the last 10 s was used in further calculations. For each chamber, three consecutive inlet–outlet pairs were measured and the seven chambers were measured sequentially. We calculated CO_2_ (*A* ^C^) and COS (*A* ^S^) net assimilation rates from the inlet and outlet concentration difference as follows:

(1) $$A=\frac{f(C_{e}-C_{o})}{M_{d}}$$

where *f* (mol s^−1^) is the inlet flow rate (dry air basis), *C*_e_ and *C*_o_ are the CO_2_ or COS mixing ratios (mol mol^−1^) entering and leaving the chamber in dry air and *M*_d_ is the sample dry mass (kg). Because mixing ratios were determined on a dry air basis (because of the Nafion dryer upstream of the QCLS) only the flow of dry air on the inlet of the chamber was necessary to perform the mass balance. Net assimilations (*A* ^C^ and *A* ^S^) were calculated from inlet–outlet pairs and then averaged (*n *=* *3) for consecutive pairs of the same chamber. The LRU rates of *A* ^C^ and *A* ^S^ normalised to their ambient concentrations were then computed as (Stimler *et al*., 2010):

(Eqn 2) $$\text{LRU}=A^{S}C_{o}^{C}/A^{C}C_{o}^{S}$$

Outlet water vapour concentration was measured with an infrared gas analyser (IRGA, LI‐6262; LI‐COR, Lincoln, NE, USA). Analyser calibration was made before the experiment with a dew‐point generator (LI‐610; LI‐COR). Outlet water vapour concentration (*W*) of each chamber was measured for 240 s, after having flushed the instrument for 600 s, and the mean of the last 10 s (*W*_o_, mol mol^−1^) was used for further calculations. The instantaneous transpiration rate of each chamber was calculated as in Eqn 1, substituting *W* for *C*, with *W*_e_ = 0. We fitted a spline to transpiration over time to derive continuous values of instantaneous transpiration for each sample (*E*_t_ in mm s^−1^ kg^−1^). The estimated cumulative transpiration of each sample (*E*_cum_ in mm kg^−1^) was then calculated as:

(Eqn 3) $$E_{\mathrm{cum}}=\sum_{i=0}^{i=n}E_{t,i}\left(t_{i}-t_{i-1}\right)$$

where *t* is time in seconds since the start of the experiment and *n* is the experiment duration. We then calculated the fresh mass (*M*_f_) of each sample at any given point in time (*M*_f,*t*_) as:

(Eqn 4) $$M_{f,t}=M_{f,\mathrm{end}}+E_{\mathrm{total}}-E_{\mathrm{cum},t}$$

where *M*_f,end_ is the sample mass at the end of the experiment and *E*_total_ is total transpiration (i.e. maximum *E*_cum_). Then, we calculated sample relative water content over time (RWC_*t*_):

(Eqn 5) $${\text{RWC}}_{t}=\frac{M_{f,t}-M_{d}}{M_{d}}100.$$

### Experimental design

We performed four experiments to: (1) characterise *A* ^C^ and *A* ^S^ during desiccation in the light and in the dark, (2) determine whether a COS compensation point and a COS source term could be detected, (3) evaluate the temperature sensitivity of any COS source term and (4) test for the effect of light intensity and temperature on COS uptake. Metabolite concentrations and gas‐exchange dynamics during desiccation in the light and in the dark were characterised for both the moss, *S. purum*, and the liverwort, *M. polymorpha* (Expt 1), whilst COS, temperature and light curves were performed only for the liverwort (Expts 2–4), with larger uptake rates per unit of dry mass.

#### 1 Desiccation curves

We measured *A* ^C^ and *A* ^S^ during desiccation for 10–13 h, in moss and liverwort samples, in the light and in the dark (Fig. S2). We ran desiccation curves separately for each bryophyte (moss and liverwort) and light–temperature regime (light on : light off, 21°C : 16°C). During each desiccation experiment, six trays with fully hydrated samples were placed into gas‐exchange chambers whilst one empty tray (also containing a temperature data‐logger) was placed into a seventh (blank) chamber to check for any COS contamination during the experiment. During the desiccation experiment the chambers were placed in a light regime with a photosynthetic photon flux density (PPFD) of 580 μmol m^−2^ s^−1^ supplied by fluorescent lamps (BriteGro 2084; Sylvania, BioSystems, Wageningen, the Netherlands). According to the manufacturer, the spectral power distribution of these lamps was 400–700 nm, with only two minor peaks detected below 400 nm (UV), of a magnitude 10 times smaller than the peaks at all other wavelengths. The temperature of the climatically controlled chamber was 16°C in the dark and 21°C in the light. Temperature inside the blank chamber was 1–2°C higher than that of the climatically controlled chamber (Fig. S3). Sample chamber temperature varied with hydration status and transpiration rate (Fig. S2). CO_2_ and COS mixing ratios (*C* ^C^ and *C* ^S^) of the inlet air were set to 410 μmol mol^−1^ CO_2_ and 540 pmol mol^−1^ COS.

#### 2 COS curves for determination of the COS‐compensation point (Γ^S^)

To test for the existence of a COS‐compensation point (Γ^S^) in the liverwort, we measured *A* ^C^ and *A* ^S^ at varying COS concentrations, in the light at 21°C and in the dark at 16°C. To minimise the effect of drying, COS curves were limited to the plateau of the *A*–RWC curve. We sequentially measured *A* ^C^ and *A* ^S^ at five *C* ^S^ values (510, 285, 385, 105 and 5–10 pmol mol^−1^) on four liverwort samples, while *C* ^C^ was kept constant (410 μmol mol^−1^). The same four samples were measured in the light and in the dark. We then estimated Γ^S^ as the *C* ^S^ at which *A* ^S^ = 0.

#### 3 Temperature response curves

To assess the effect of temperature on the COS source term (*P* ^S^), we performed additional COS curves at three chamber temperatures (16, 21 and 25°C), in the light and in the dark. We measured four liverwort samples for each combination of temperature set point and light regime (for 25 and 16°C in the light and 16°C in the dark, only three). We measured *A* ^C^ and *A* ^S^ at four *C* ^S^ (120, 200, 400 and 520 pmol mol^−1^) while *C* ^C^ was kept constant (410 μmol mol^−1^). For each sample, including those from Expt 3, we estimated *P* ^S^ as the intercept of the linear regression between *A* ^S^ and *C* ^S^, that is, *A* ^S^ at *C* ^S^ = 0.

#### 4 Light and temperature curves

To assess the effect of light intensity and temperature on COS uptake and emission, we measured *A* ^C^ and *A* ^S^ whilst gradually increasing light intensity and temperature. Similar to the COS curves, to minimise the effect of RWC, we successively measured *A* ^C^ and *A* ^S^ at five PPFD values (0, 90, 255, 420 and 580 μmol m^−2^ s^−1^), within the plateau of the *A* ^S^–RWC curve. Light curves were performed under ambient *C* ^C^ and *C* ^S^ (410 μmol mol^−1^ and 510 pmol mol^−1^, respectively) on four liverwort samples and under ambient *C* ^C^ and near‐zero *C* ^S^ (5–10 pmol mol^−1^) on four different samples. The temperature inside the gas‐exchange chamber increased with light intensity (Fig. S3).

### Biochemical assays

To assess the change in total protein and nonstructural carbohydrate (NSC) content during desiccation we performed additional desiccation curves, under similar conditions as described earlier, in the light and in the dark, with successive sampling. We collected three replicate samples (per species and light level) consisting of 1–3 g of tissue at five points in time. For the liverworts, tissue RWC at different points in time was estimated from three independent samples (for each light level) that were conserved intact along the whole desiccation curve. For the mosses, tissue RWC was measured individually on a separate sub‐sample at the time of collection.

Quantification of total protein content and NSC was performed following enzymatic digestion as in Biais *et al*. (2014) at the HitMe platform of the INRA‐Bordeaux Metabolome Facility (France). Briefly, *c*. 20 mg aliquots of frozen sample were powder‐homogenised and fractionated three times at 95°C for 15 min with 250 and 150 μl (80% v/v) and 250 μl (50% v/v) ethanol, 10 mM Hepes/KOH (pH 6). Glucose, fructose and sucrose concentrations were quantified from the ethanolic supernatant following an adapted procedure from Jelitto *et al*. (1992). Aliquots of 50 μl of ethanolic extract were added to 160 μl of a mix of 100 mM Hepes–KOH buffer (pH 7), 3 mM MgCl_2_, 3 mM ATP, 1.3 mM NADP and five units of glucose‐6‐phosphate dehydrogenase. Then, one unit of hexokinase, one unit of phosphoglucose isomerase and 30 units of invertase were added successively. Glucose, fructose and sucrose contents were quantified from the difference in absorbance between successive steps. Total protein content and starch were determined from the pellet resuspended in 100 mM NaOH and heated at 95°C for 30 min (Hendriks *et al*., 2003). Total protein content was quantified using Bradford reagent (Bradford, 1976). Analyses were run in duplicate. Extractions and assays were performed using a robotised Starlet platform (Hamilton, Villebon sur Yvette, France) and absorbancies were read at 340 nm for NSC and 600 nm for protein in MP96 readers (SAFAS, Monaco). All chemicals were purchased from Sigma‐Aldrich Ltd (Gillingham, UK) and enzymes from Roche Applied Science (Meylan, France).

### Statistical analyses

To test that *A* ^S^ would show an optimum‐like response to desiccation similar to that of *A* ^C^ in the light, and that *A* ^S^ during desiccation would not differ between the light and the dark, we fitted general additive mixed models (GAMMs). GAMMs were fitted to *A* ^C^ and *A* ^S^ with RWC as a predictor and taking into account the random sample‐to‐sample variability (Wood, 2006). Significant differences (α = 0.05) between the light and the dark were assessed graphically based on nonoverlapping 95% confidence intervals (Cis). We used the package mgcv in R v.3.3.1 (R Core Team, 2014). To quantify the effect of *C* ^S^ on liverwort *A* ^C^ and *A* ^S^, we fitted linear mixed models (LMMs) with *C* ^S^ and light and temperature regime (dark/cool vs light/warm) as fixed predictors and sample as random factor, using the packages lme4 and lmertest and investr, to calculate Γ^S^ (Greenwell & Schubert Kabban, 2014). We obtained the COS source term (*P* ^S^) as the intercept of the *A* ^S^–*C* ^S^ relationships measured at varying temperatures, in the light and in the dark. Since *P* ^S^ increased exponentially with temperature, we fitted a linear relationship between log_e_‐transformed *P* ^S^ and temperature and then calculated the relative increase per 10°C increase (*Q*_10_) as in Eqn 3 in Zaragoza‐Castells *et al*. (2007). To analyse the effect of PPFD and COS availability on *A* ^S^ we fitted an LMM with PPFD and source air (ambient vs near‐zero COS) as fixed predictors and the sample as a random factor. In our experimental setup, the increase in light intensity was coupled to a 5°C increase (Fig. S3), potentially affecting both COS uptake and emission, irrespective of light intensity. To partially disentangle the effects of temperature on COS uptake and emission, we first estimated the *P* ^S^ for each measurement from temperature and the *Q*_10_ and then calculated the gross COS uptake (*U* ^S^) as the sum of the net uptake and the source. Finally, we performed a similar LMM on estimated *U* ^S^ with PPFD and source air as fixed predictors. For photosynthesis, the *A* ^C^ shows a response to PPFD of the form:

(Eqn 6) $$A^{C}=R_{d}+A_{\max}^{C}\left(1-e^{-k\mathrm{PPFD}}\right)$$

where *R*_d_ is the net CO_2_ emission in the dark, *A*_max_ is the asymptote of the curve and *k* is a constant, such that *A*_max_ *k* is the slope of the initial part of the curve (Marschall & Proctor, 2004). We fitted Eqn 6 to *A* ^C^–PPFD measurements and tested for differences between ambient and near‐zero *C* ^S^ by comparing the 95% CI of the fitted parameters. Finally, to assess the effect of light on total protein and NSC during desiccation, we performed linear model analyses with RWC, light and their interaction on metabolite concentrations. Before analyses, we checked for normality and log_e_‐transformed metabolite concentrations and RWC (the latter for mosses only).

## Results

### COS uptake dynamics along desiccation in the light and in the dark and LRU

**Figure 1:**
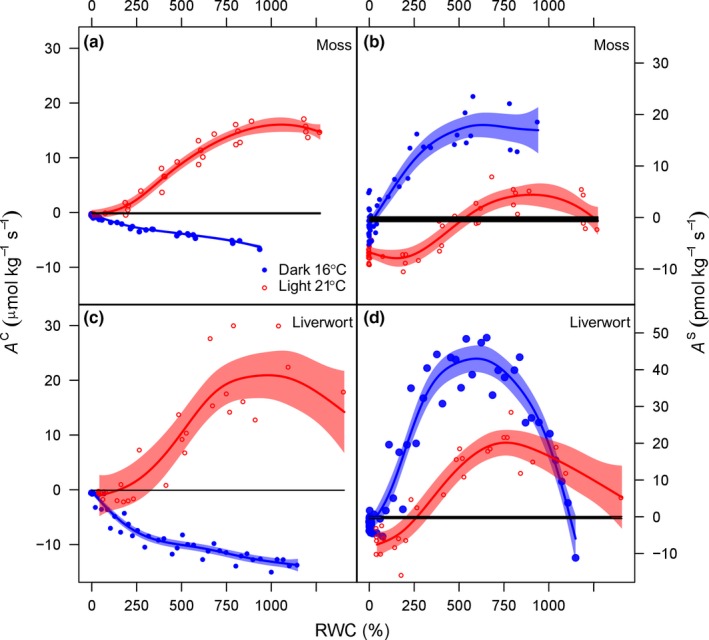
(a, c) CO_2_ (*A*^C^) and (b, d) carbonyl sulphide (COS) (*A*^S^) net assimilation in the light at 21°C (red) and in the dark at 16°C (blue) with decreasing tissue relative water content (RWC) in (a, b) the moss *Scleropodium purum* and (c, d) the liverwort *Marchantia polymorpha*. Each symbol is an individual sample, the lines are smooth curves (fitted with a generalised additive model) and the blue and red areas denote the 95% confidence interval for *A*^C^ and *A*^S^ in the light (red) or in the dark (blue). Areas where the confidence interval do not overlap denote a significant effect at α = 0.05. The nonRWC‐dependent black regions (at *c*. 0) denote the mean (± SD) fluxes of the blank chamber.

The typical optimum‐like response of the net CO_2_ uptake (*A* ^C^) during dehydration in the light, characteristic of bryophytes, was clearly observed in the liverwort*, M. polymorpha* (Fig. 1c), while in the moss, *S. purum*, the increase in *A* ^C^ upon initial desiccation from maximum RWC was less evident (Fig. 1a). Maximum *A* ^C^ in the light was similar between the moss and the liverwort (15.9 and 22.7 μmol kg^−1^ s^−1^, respectively), whereas maximum *A* ^S^ was four times higher in *M. polymorpha*, the liverwort, compared with *S. purum*, the moss (20.7 ± 3.6 and 5.2 ± 2.3 pmol kg^−1^ s^−1^, estimated maximum from fitted GAMM ± 95% CI). In the dark, at a lower temperature (16°C), respiration (*A* ^C^ < 0) decreased progressively during tissue desiccation until it reached zero (Fig. 1a,c), whilst *A* ^S^ followed an optimum‐like response to desiccation similar to that observed for *A* ^C^ in the light, in both bryophytes (Fig. 1b,d). These data support our assumption that COS uptake by astomatous plants continues in the dark. Contrary to our expectations, however, *A* ^S^ in the dark was higher under the dark and cooler conditions along the whole desiccation curve, in both the liverwort, *M. polymorpha*, and the moss, *S. purum* (Fig. 1b,d). Furthermore, towards the end of the desiccation curves in the light (and at 21°C), *A* ^S^ shifted from net uptake to net emission in both species, while in the dark (and at 16°C) *A* ^S^ remained positive, or not significantly different from zero, during the entire desiccation curve (Fig. 1b,d). It is worth noting that during the desiccation experiments our blank chambers showed no signs of COS or CO_2_ uptake or release throughout. Collectively, the earlier results led to negative values of LRU not only in the dark, but also in the light when RWC fell below its optimum value for gas exchange (Table 1). At optimum RWC in the light, the calculated LRU was 0.22 for the moss and 0.89 for the liverwort.

**Table 1:** Estimated net uptake (SE) of CO_2_ (*A* ^C^ in μmol kg^−1^ s^−1^), carbonyl sulphide (COS) (*A* ^S^ in pmol kg^−1^ s^−1^) and leaflet relative uptake (LRU), in the light at 21°C and in the dark at 16°C, at the optimal (when *A* ^S^ was maximal) and minimal tissue relative water content (RWC in %) in the moss *Scleropodium purum* and the liverwort *Marchantia polymorpha*

			RWC	*A* ^C^	*A* ^S^	LRU
Moss	Optimum	Light	887	15.3 (0.6)	4.3 (1.0)	0.22
		Dark	661	−4.9 (0.2)	18.0 (1.1)	−3.08
	Minimum	Light	1	−0.3 (0.3)	−6.9 (0.5)	15.12
		Dark	1	−0.3 (0.1)	−1.3 (0.6)	3.43
Liverwort	Optimum	Light	762	18.8 (1.7)	20.2 (1.8)	0.89
		Dark	598	−10.7 (0.3)	42.9 (1.9)	−3.04
	Minimum	Light	42	−1.5 (1.8)	−7.5 (1.8)	4.14
		Dark	1	−0.4 (0.1)	−2.1 (0.7)	4.24

### COS compensation point (Γ^S^) and temperature sensitivity of source term (*P* ^S^) in the liverwort

**Figure 2:**
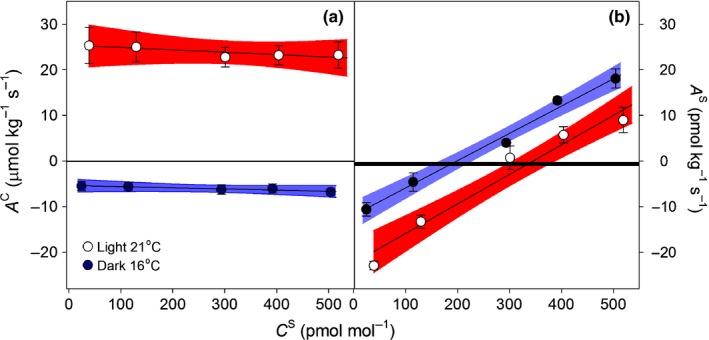
Mean (± SE,* n *=* *4) (a) CO_2_ (*A*^C^) and (b) carbonyl sulphide (COS) (*A*^S^) net assimilation along a COS mixing ratio (*C*^S^) gradient in the light at 21°C (open symbols and red areas) and in the dark at 16°C (closed symbols and blue areas), in the liverwort *Marchantia polymorpha*. Lines and areas represent linear fits (*P *<* *0.001, *R* ^2^ = 0.9 in b), and their 95% confidence intervals. Black regions denote the mean (± SD) fluxes of the blank chamber.

Both CO_2_ photosynthetic uptake in the light and CO_2_ respiratory release in the dark were unaffected by *C* ^S^ (Fig. 2a; Table S1). By contrast, and as expected, *A* ^S^ increased linearly with *C* ^S^ (*P *<* *0.001, Fig. 2b) regardless of the light and temperature regimen. LMM revealed that the rate of increase of *A* ^S^ with *C* ^S^ did not differ between the two light and temperature regimes (*P *=* *0.526, Fig. 2b), but predicted *P* ^S^ (*A* ^S^ extrapolated at zero *C* ^S^) was significantly (*P *=* *0.005) greater in the light at 21°C (21 ± 2.7 pmol kg^−1^ s^−1^) than in the dark at 16°C (12 ± 1.9 pmol kg^−1^ s^−1^). For *M. polymorpha*, we estimated a COS compensation point (Γ^S^) of 345 ± 68 pmol mol^−1^ in the light at the higher temperature and 199 ± 74 pmol mol^−1^ in the dark at the lower temperature. During all experiments manipulating COS concentrations, light intensity and temperature in our blank chamber remained constant, indicating that chamber artefacts such as COS emission or uptake were minimised in our experimental set‐up despite large changes in environmental conditions.

**Figure 3:**
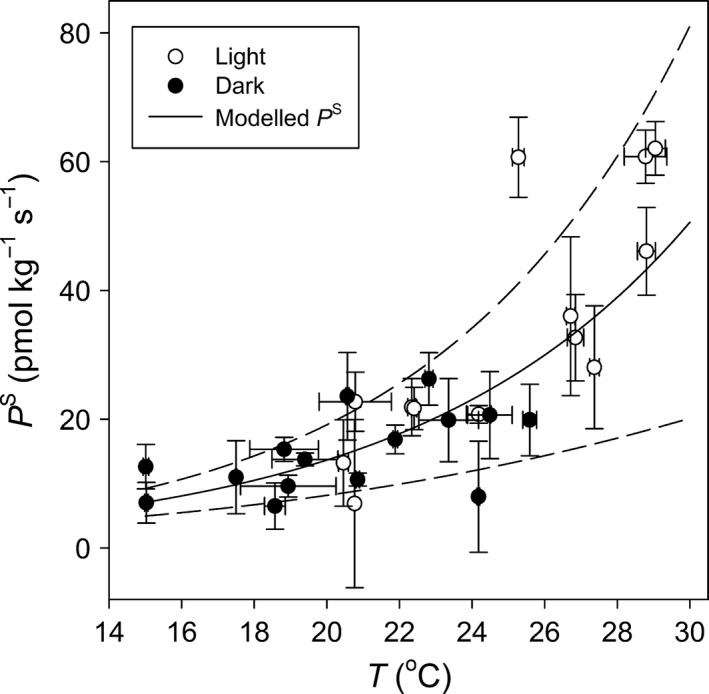
Relationship between mean measurement temperature (*T*,* n *=* *4 measurements) and estimated (± SE) carbonyl sulphide (COS) emission (*P*^S^) from the intercept of *A*^S^–*C*^S^ curves (*A*^S^ is net COS assimilation and *C*^S^ is the COS mixing ratio), in the liverwort *Marchantia polymorpha*. Open and closed symbols represent estimated *P*^S^ from *A*^S^–*C*^S^ curves performed in the light or in the dark, respectively. The lines represent the modelled common temperature response (continuous line) and its uncertainty (dashed lines) over the experimental temperature range: modelled $P^{S}=e^{\frac{\log(Q_{10})}{10}}$ with *Q*_10_ = 3.7

Our estimates of *P* ^S^ (*A* ^S^ at zero *C* ^S^) represent a COS emission rate coming from the liverwort. We found that *P* ^S^ increased exponentially with temperature (*t *=* *6.5, *P *<* *0.001), regardless of the light/dark regime with a *Q*_10_ of 3.7 (Fig. 3).

### Effect of light and temperature on net (*A* ^S^) and gross (*U* ^S^) COS uptake

**Figure 4:**
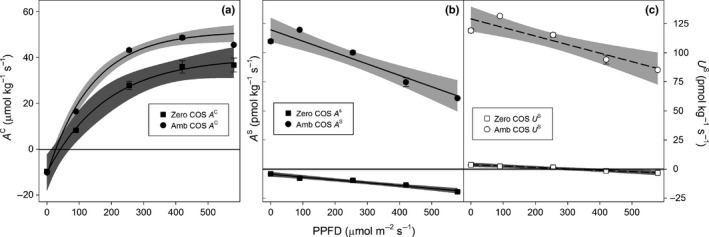
Relationship between light intensity (photosynthetic photon flux density, PPFD) and mean (± SE,* n *=* *4) net measured (a) CO_2_ (*A*^C^) and (b) carbonyl sulphide (COS) assimilation (*A*^S^, closed symbols and solid lines) and (c) gross estimated assimilation (*U*^S^, open symbols and dashed lines) under ambient COS mixing ratio (Amb COS, squares) and near‐zero COS mixing ratio (Zero COS, circles), in the liverwort *Marchantia polymorpha*. Lines and grey areas correspond to the (a) exponential and (b, c) linear fits and their 95% confidence interval. Black regions denote the mean (± SD) fluxes of the blank chamber.

In *M. polymorpha*,* A* ^C^ increased with increasing light intensity and temperature, until it reached a plateau (Fig. 4) according to Eqn 4. We found that the *A* ^C^–PPFD relationship did not change between ambient and near‐zero COS supply (Fig. 4a), as demonstrated by the overlap of the 95% CI of the parameter estimates (Table S1). By contrast, *A* ^S^ decreased with increasing light intensity and temperature, under both ambient and near‐zero COS supply, but the rate of change with light intensity was lower under near‐zero than ambient *C* ^S^ (Fig. 4b; Table S1). This decrease in *A* ^S^ with light and temperature is partly explained by the temperature dependence of *P* ^S^. However, even after accounting for the temperature effect on *P* ^S^, we found that the gross COS uptake (*U* ^S^ = *A* ^S^ + *P* ^S^) still decreased with light intensity under ambient COS (Fig. 3c; Table S1). This could suggest an inhibitory effect of light intensity on COS uptake. However, we cannot completely discard the influence of an experimental artefact potentially biasing our observations. For example, given our experimental sequence, uptake rates at the highest light intensities were measured towards the end of the experiment, when decreasing RWC could have negatively affected uptake.

### Total protein and NSC accumulation in the light and in the dark along desiccation

**Figure 5:**
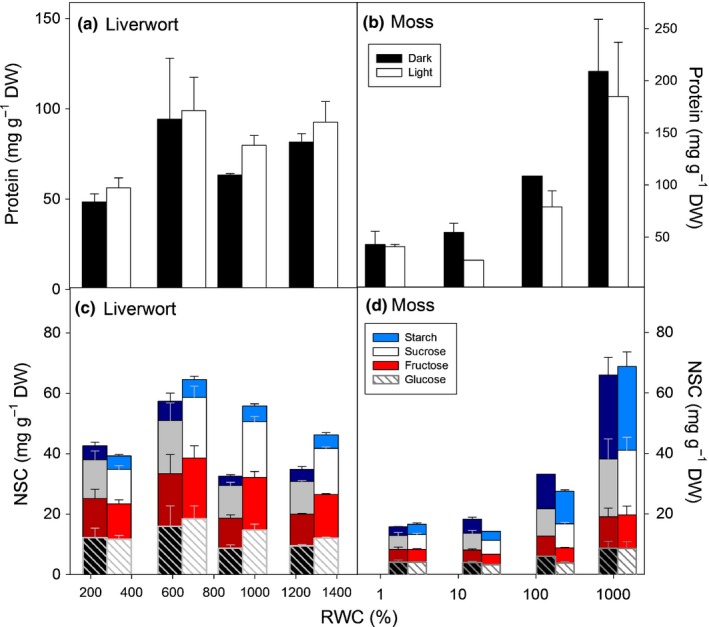
(a, b) Protein and (c, d) total nonstructural carbohydrate (NSC) content, per unit of dry weight (DW), in (a, c) the moss *Scleropodium purum* and (b, d) the liverwort *Marchantia polymorpha* measured along decreasing relative water content (RWC) in the dark (closed bars) and in the light (open bars). Bars are the means (+ SE,* n *=* *1–5) for grouped data according to four categorical levels of RWC (very high, high, intermediate and low), RWC values on the *x*‐axis are the overall means for the light and dark treatments for each RWC level.

Total protein content decreased with desiccation in both the liverwort (*M. polymorpha*) and the moss (*S. purum* Table 2). In the moss, NSCs and all their components (glucose, fructose, sucrose and starch) also decreased with desiccation (Figs 5, S4; Table 2). The decreased rate with RWC of protein and NSCs (in the moss) with desiccation did not differ between the light and the dark (Table 2).

**Table 2:** Results of the linear model (*t* and *P*) to assess the effect of relative water content (RWC), light and temperature regime (light : dark, 21°C : 16°C) and their interaction on different metabolite concentrations (proteins and nonstructural carbohydrates, NSCs) in the liverwort *Marchantia polymorpha* and the moss *Scleropodium purum*. Significant effects (*P* < 0.05) are indicated in bold

	Metabolite	RWC		Light		RWC × Light	
		*t*	*P*	*t*	*P*	*t*	*P*
Liverwort	Glucose	−0.597	0.556	0.319	0.752	0.529	0.601
	Fructose	−0.591	0.56	0.084	0.934	0.932	0.36
	Sucrose	−0.416	0.681	0.008	0.994	1.047	0.305
	Starch	−0.626	0.536	−0.095	0.925	0.661	0.514
	Total NSCs	−0.533	0.599	0.161	0.873	0.798	0.432
	Protein	2.267	**0.032**	1.036	0.31	−0.241	0.812
Moss	Glucose	2.485	**0.022**	0.28	0.782	−0.336	0.74
	Fructose	2.752	**0.013**	0.282	0.781	−0.081	0.936
	Sucrose	3.953	**< 0.001**	0.381	0.708	0.241	0.812
	Starch	10.077	**< 0.001**	0.842	0.41	−0.3	0.767
	Total NSCs	5.458	**< 0.001**	0.512	0.615	−0.115	0.91
	Protein	5.262	**< 0.001**	0.18	0.859	−0.383	0.706

## Discussion

### Challenging the unidirectional flux assumption for the vegetation COS flux

Here, we aimed to critically assess some of the key assumptions underlying the relationship between COS and CO_2_ uptake in bryophytes. We hypothesised that COS uptake rates in bryophytes would be light‐independent. Our results on two astomatous bryophytes (one moss and one liverwort) partially agreed with this prediction as we found that net COS uptake (*A* ^S^) in the dark remained positive, and this supports the current idea that COS hydrolysis is dominated by the enzyme CA, which is assumed to be light‐independent (Protoschill‐Krebs *et al*., 1996). However, our results also showed that *A* ^S^ may be affected by light in an unexpected way. We found that for an equivalent hydration status, *A* ^S^ was significantly greater in the dark than in the light. A plausible explanation for this observation is that bryophyte *A* ^S^ is the net result of two opposing fluxes, uptake and emission, with COS emission being of greater magnitude at higher temperatures in the light than in cooler dark conditions. All our other results are compatible with this explanation. First, we observed that below an optimal hydration status, *A* ^s^ shifted from net uptake to net emission in the light, but not in the dark. Also *A* ^S^ decreased with increasing light intensity and higher temperatures. Finally, we found a nonzero compensation point (Γ^S^) that was greater in the light than in the dark. Previously, Kuhn & Kesselmeier (2000) had suggested the existence of a nonzero Γ^S^ in lichens. Nonzero Γ^S^ values have also been observed in some higher plants (Kesselmeier & Merk, 1993) but their influence on the overall COS uptake rate seemed small (Seibt *et al*., 2010; Stimler *et al*., 2010). These observations, together with other studies conducted on plants senescing, under fungal attack or under heat and drought stress (Bloem *et al*., 2012; Maseyk *et al*., 2014; Commane *et al*., 2015), suggest that plant COS emissions may be more ubiquitous than previously assumed. Our results encourage further studies revisiting COS fluxes from vascular plants, for example performing *A* ^S^–*C* ^S^ curves at different temperatures, to determine whether COS emissions can be detected with the new generation of commercially available COS analysers offering much higher precision (*c*. 5 ppt). In particular, experiments using mutants (Costa *et al*., 2015) that maintain stomata open in the dark could provide a novel approach to detect COS emissions from vascular plants and how the gross COS fluxes respond to different environmental drivers.

The existence of a bi‐directional COS flux contradicts our initial expectation for COS uptake by astomatous bryophytes, because there are currently no described leaf‐level processes that result in COS as a byproduct (Protoschill‐Krebs *et al*., 1996; Bloem *et al*., 2015). Previously, Fried *et al*. (1993) measured COS emissions in the light from a peat soil and moss microcosm, but they ascribed the emissions to the soil component. Indeed, Whelan & Rhew (2015) demonstrated that soils can emit COS and that the rate of COS emission increases in the light and with higher temperatures. Whelan & Rhew (2015) suggested that COS originated from abiotic photo‐degradation of dead organic matter by UV light, similar to COS emissions measured from sea, lake and precipitation water (Zepp & Andreae, 1994; Mu *et al*., 2004; Du *et al*., 2017). However, based on the manufacturer's specifications for our light source (see the Materials and Methods section) and because our chambers were not made of UV‐transparent quartz glass, we assume that our bryophyte samples were not exposed to high‐intensity UV radiation. Thus it is unlikely that our COS emissions in the light would have been strongly affected by UV‐driven organic matter degradation. Our results suggest that an additional light‐independent process of biological origin underlies observed COS emission from bryophytes. We argue that this process is likely to be of biological origin because, in the dark, COS emission at minimum hydration status (below 70% RWC) was not detectable, while our *A* ^S^–*C* ^S^ curve demonstrated that COS emission still occurred in the dark at optimal hydration status. Despite uncertainties in the mechanistic driver of the emissions, in our study we observed protein degradation during desiccation in the moss and below optimal hydration in the liverwort that could have led to the liberation and eventual catabolism of sulphur‐containing amino acids (cysteine and methionine), potential precursors for COS production (Zepp & Andreae, 1994; Bloem *et al*., 2004; Mu *et al*., 2004; Du *et al*., 2017). This protein degradation would have occurred while the leaflet tissues were metabolically active and was accompanied by a significant decrease in NSC content. This result alongside the gas exchange data suggest that as the leaflets became progressively water‐stressed, protein degradation affected the photosynthetic machinery, including CA, and probably caused the increased apparent COS emission by reducing gross COS uptake. This is consistent with results in the literature on vascular plants that have shown that during water stress the total protein content decreases rapidly and is accompanied by a strong reduction in the activity of key enzymes involved in carbon assimilation (Majumdar *et al*., 1991; Khanna‐Chopra, 2012). The metabolic activity of accompanying microorganisms, sensitive to water stress too (Vacher *et al*., 2016), could also be contributing to the observed COS emission in bryophytes. Bryophytes, like any other plants, host rich microbial communities and both liverworts and feather‐like mosses are known to form symbiotic associations with fungi and bacteria (DeLuca *et al*., 2002; Humphreys *et al*., 2010; Davey *et al*., 2012). It has recently been shown that some fungal and bacterial enzymatic reactions produce COS (Masaki *et al*., 2016; Ogawa *et al*., 2016); hence it is plausible that natural symbionts could also be contributing to the net COS fluxes in bryophytes.

### Assessing the climatic sensitivity of COS uptake and emission

In bryophytes, tissue water content is the main driver of net CO_2_ exchange (*A* ^C^, Wagner *et al*., 2013) and we expected the same for net COS uptake (*A* ^S^). We indeed observed that *A* ^S^ was strongly driven by tissue water content, but our results also showed that *A* ^S^ was sensitive to temperature. Our observations on liverworts in the dark showed that *A* ^S^ at optimal water content was lower at 21°C than at 16°C, while CO_2_ respiratory release was greater at the higher temperature (Fig. S5). This seems to contradict the increase of *A* ^S^ observed in lichens by Kuhn & Kesselmeier (2000) for the same temperature range. Since a thermal optimum below 21°C for enzymatic COS hydrolysis is not within the range of published data (Burnell & Hatch, 1988), we argue that lower net *A* ^S^ at a higher temperature (within our measurement range) is caused by higher COS emissions rather than by reduced COS uptake. In fact, here we demonstrated that the COS source term (*P* ^S^, estimated from *A* ^S^–*C* ^S^ curves) is very sensitive to temperature, with a *Q*_10_ of 3.7. Our *Q*_10_ estimate for COS emissions is higher than those reported for net COS fluxes in soils (Maseyk *et al*., 2014) but within the range of respiratory *Q*_10_ for several moss species (Wagner *et al*., 2013).

Our results also seem to indicate a small, but statistically significant, decrease of the gross COS uptake (*U* ^S^) with increasing light intensity. Given our current knowledge of the temperature sensitivity of CA activity (Burnell & Hatch, 1988), it is unlikely that COS hydrolysis was inhibited by the warming experienced inside the gas‐exchange chamber during our light curves. It could be argued that this decrease in *U* ^S^ with light intensity was simply driven by a reduction in tissue RWC along the experiment. In our experiment, for light intensities above 400 μmol m^−2^ s^−1^ the mean RWC was 535 ± 25%, that is, close to the point beyond which *A* ^S^ starts to decrease. Yet, such reduction in RWC should have also negatively affected *A* ^C^ and we did not detect a major drop in *A* ^C^ towards the end of the light curve. Alternatively, other biological reasons may explain this decrease of *U* ^S^ with light intensity in bryophytes. For example, carbon concentration mechanisms (CCMs) that incorporate CA as a key constituent have evolved in some bryophyte lineages (specifically in the Anthocerophyta, the hornworts), but there is no conclusive evidence from previous gas exchange measurements for an active CCM in either mosses or liverworts (Smith & Griffiths, 2000; Badger, 2003; Meyer *et al*., 2008). However, the presence of CAs of different types associated with a basal CCM has been hypothesised in all C_3_ plants (Zabaleta *et al*., 2012), and thus one hypothesis that might deserve future study would be to test whether the observed decrease in *A* ^S^ with light could be explained by activity of a light‐sensitive CA (Rech *et al*., 2008). However, in one of our study species (*M. polymorpha*, the liverwort), the photosynthetic CO_2_ compensation point and carbon isotope discrimination did not respond to the addition of a CA‐inhibitor, indicating that it probably lacks any external CA or CCM activity (Smith & Griffiths, 2000).

### The unexpected contribution of bryophytes to the COS budget

The quantification of COS fluxes in bryophytes is not only relevant to better understand the drivers of the net leaf COS flux in other species, but also to help constrain the global COS budget, as bryophytes are key constituents of many ecosystems (DeLuca *et al*., 2002). The estimated leaflet relative uptake rates (LRU of 0.2 for the moss, *S. purum*, and 0.9 for the liverwort, *M. polymorpha*, in the light and at optimal hydration status) found in our study were lower than current LRU estimates for vascular plants, which range between 1.4 and 2 (Seibt *et al*., 2010; Stimler *et al*., 2010). If we were to estimate the contribution of cryptogamic covers to the global COS budget from their current estimates of CO_2_ uptake (3.9 Pg C yr^−1^ according to Elbert *et al*., 2012) following the same LRU approach as proposed for vascular plants (Sandoval‐Soto *et al*., 2005; Campbell *et al*., 2008), with atmospheric mixing ratios of 400 μmol CO_2_ mol^−1^ and 540 pmol COS mol^−1^, we would arrive at an estimate between 0.005 and 0.024 Tg COS yr^−1^ (for LRU of 0.2 and 0.9, respectively). This flux is within the same order of magnitude as the estimate of COS uptake for swamps and marshes and is larger than current estimates for tundra, alpine and desert scrublands (see table 3 of Sandoval‐Soto *et al*., 2005). These values could serve as first approximations for high latitudes in the summer, where extensive regions are dominated by uniform bryophyte carpets, daylight is continuous and evaporative demand is low (Lindo *et al*., 2013). However, bryophytes are also commonly found in areas where day–night cycles alternate and physiological activity is strongly constrained by tissue hydration (Elbert *et al*., 2012). In these areas, during the day, when temperatures are high and air moisture is low, bryophytes would tend to dehydrate and CO_2_ and COS uptake would be metabolically limited, whilst higher temperatures and incident radiation would enhance unexpected COS emission. By contrast, at night, when the temperature is lower and the evaporative demand is low, bryophytes would rehydrate towards full turgor and act as strong COS sinks but CO_2_ sources. Our estimated *Q*_10_ constitutes a first step towards quantifying the contribution of the COS emissions. However, further work is required to understand the sensitivity of this parameter (*P* ^S^) to additional environmental constraints, particularly changes in tissue hydration and light regimes with seasons or ontogeny (Porada *et al*., 2013).

### Conclusions

Here, using bryophytes as model organisms, we have demonstrated that net COS uptake continues in the dark, but is also progressively decreased as irradiance and temperature increase, mostly because of an unexpected, temperature‐driven COS emission. Together, our results challenge a key underlying assumption for quantifying GPP from COS fluxes that vegetation COS uptake is unidirectional. Obviously, we cannot immediately extrapolate our findings to other terrestrial vascular plants; however, our results should encourage further studies to revisit the unidirectional flux assumption in vascular plants making use of the improved laser spectrometers now available.

## Author contributions

T.E.G., L.W., J.O. and J.R. conceived and designed the experiment. T.E.G., S.P.J., R.B., S.W., J.S. and J.B.W. designed and took the gas‐exchange measurements. T.E.G., C.B. and Y.G. performed and interpreted the biochemical analyses. T.E.G., J.O., J.B.W. and L.W. analysed the data. T.E.G. wrote the first manuscript draft. J.O., J.R., Y.G., J.B.W., R.B., S.P.J., J.S., S.W., C.B., B.G. and L.W. commented and contributed to the final version.

## Supporting information

Please note: Wiley Blackwell are not responsible for the content or functionality of any Supporting Information supplied by the authors. Any queries (other than missing material) should be directed to the *New Phytologist* Central Office.

**Fig. S1** Allan variance plot showing the standard deviation for the QCLS.
**Fig. S2** Tissue relative water content and sample temperature with desiccation.
**Fig. S3** Sample temperature inside the gas‐exchange chamber during light curves.
**Fig. S4** Individual metabolite (protein and nonstructural carbohydrate) concentrations.
**Fig. S5** CO_2_ and COS net uptake rates in the dark with desiccation at two temperatures.
**Table S1** Estimated regression coefficients and summary statistics of the linear mixed models performed to assess the effects of COS concentration in the light and in the dark, and light intensity under ambient and near‐zero COS mixing rations on CO_2_ and COS uptake rate
