# Supplementary material for: Bryophyte gas‐exchange dynamics along varying hydration status reveal a significant carbonyl sulphide (COS) sink in the dark and COS source in the light
Source: New Phytol. 2017 May 3;215(3):965–76. doi: 10.1111/nph.14584 (PMC5518222; doi:10.1111/nph.14584)
Supplement: Supplementary file 1 — Fig. S1 Allan variance plot showing the standard deviation for the QCLS. Fig. S2 Tissue relative water content and sample temperature with desiccation. Fig. S3 Sample temperature inside the gas‐exchange chamber during light curves. Fig. S4 Individual metabolite (protein and nonstructural carbohydrate) concentrations. Fig. S5 CO2 and COS net uptake rates in the dark with desiccation at two temperatures. Table S1 Estimated regression coefficients and summary statistics of the linear mixed models performed to assess the effects of COS concentration in the light and in the dark, and light intensity under ambient and near‐zero COS mixing rations on CO2 and COS uptake rate [file NPH-215-965-s001.pdf]

New Phytologist

***New Phytologist* Supporting Information**

Article title: Bryophyte gas-exchange dynamics along varying hydration status reveal a significant COS sink in the dark and COS source in the light

Authors: Teresa E. Gimeno, Jérôme Ogée, Jessica Royles, Yves Gibon, Jason B. West, Régis Burlett, Sam P. Jones, Joana Sauze, Steven Wohl, Camille Benard, Bernard Genty, Lisa Wingate

Article acceptance date: 21 March 2017

The following Supporting Information is available for this article:

Fig. S1 Allan variance plot showing the standard deviation for the QCLS.

Fig. S2 Tissue relative water content and sample temperature along desiccation.

Fig. S3 Sample temperature inside the gas-exchange chamber during light curves.

Fig. S4 Individual metabolite (protein and non-structural carbohydrate) concentrations.

Fig. S5 CO_2 and COS net uptake rates in the dark along desiccation at two temperatures.

Table S1 Estimated regression coefficients and summary statistics of the linear mixed models performed to assess the effects of COS concentration in the light and in the dark, and light intensity under ambient and near-zero COS mixing rations on CO_2 and COS uptake rate

Fig. S1 Allan variance plot showing the standard deviation for the COS mixing ratio (in nmol mol^{-1} or ppb) measured with the quantum cascade laser spectrometer (QCLS) over time

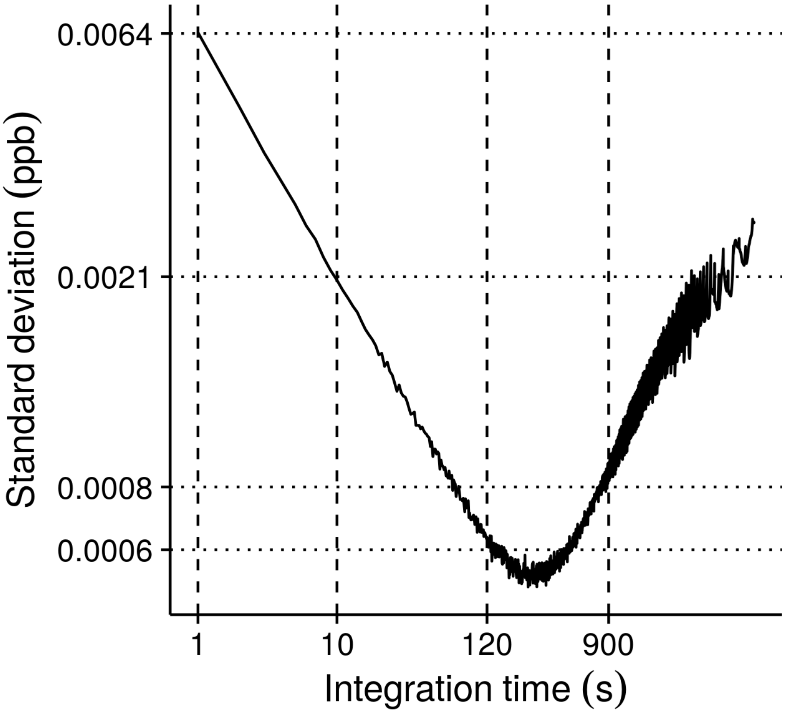

Fig. S2 Decreasing tissue relative water content (RWC upper panels) and increasing temperature (*T*, lower panels) in the liverwort (*Marchantia polymorpha*, left) and the moss (*Scleropodium purum*, right), in the light at 21 °C (open, dashed lines) and in the dark at 16 °C (closed symbols, continuous lines), over time. RWC decreased following a negative exponential function of the form: RWC = $RWC_0 + a\ e^{(-b\ time)}$, where RWC_0 is the estimated RWC at the start of the gas-exchange measurements and a and b are fitted coefficients. All four non-linear regressions fitted to the observations were significant ($P < 0.001$) with $R^2 \geqslant 0.9$

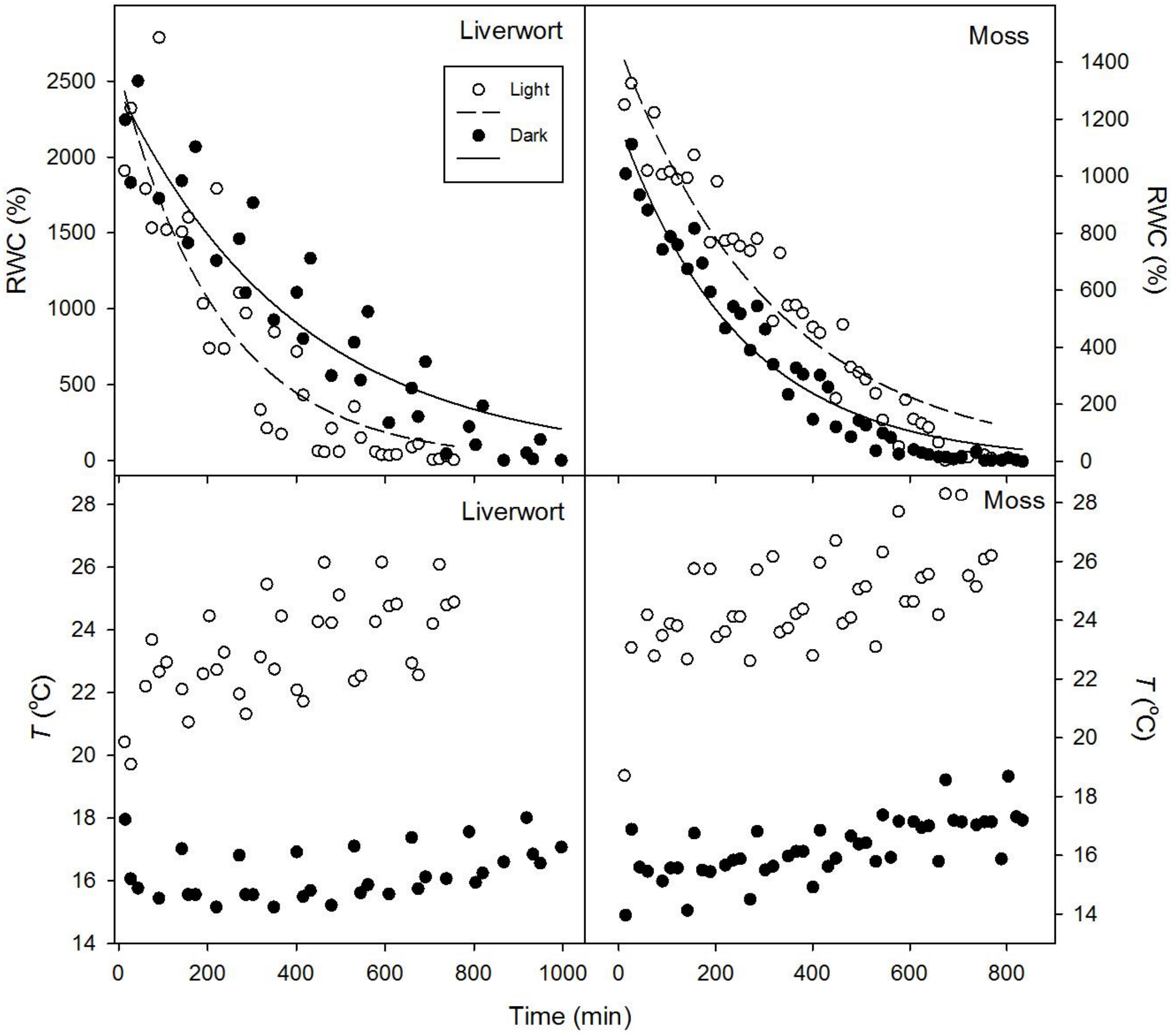

Fig. S3 Change in temperature (*T*) coupled to increasing photosynthetic photon flux density (PPFD) along a light curve, inside the glass-chambers with (sample) and without (blank) liverwort (*M. polymorpha*) samples

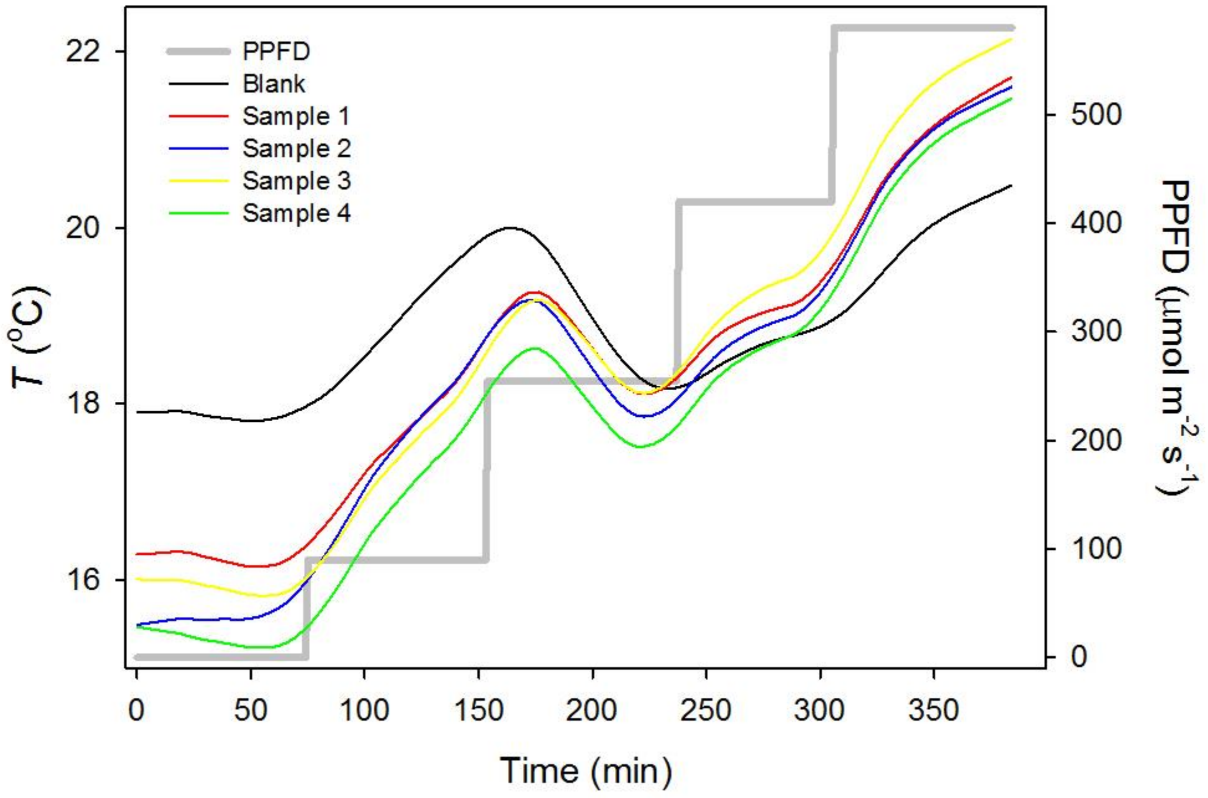

Fig. S4 Individual metabolite concentrations per unit of dry weight (DW) in the light (open) and in the dark (closed symbols) with decreasing tissue relative water content (RWC) in the liverwort (*Marchantia polymorpha*) and in the moss (*Scleropodium purum*)

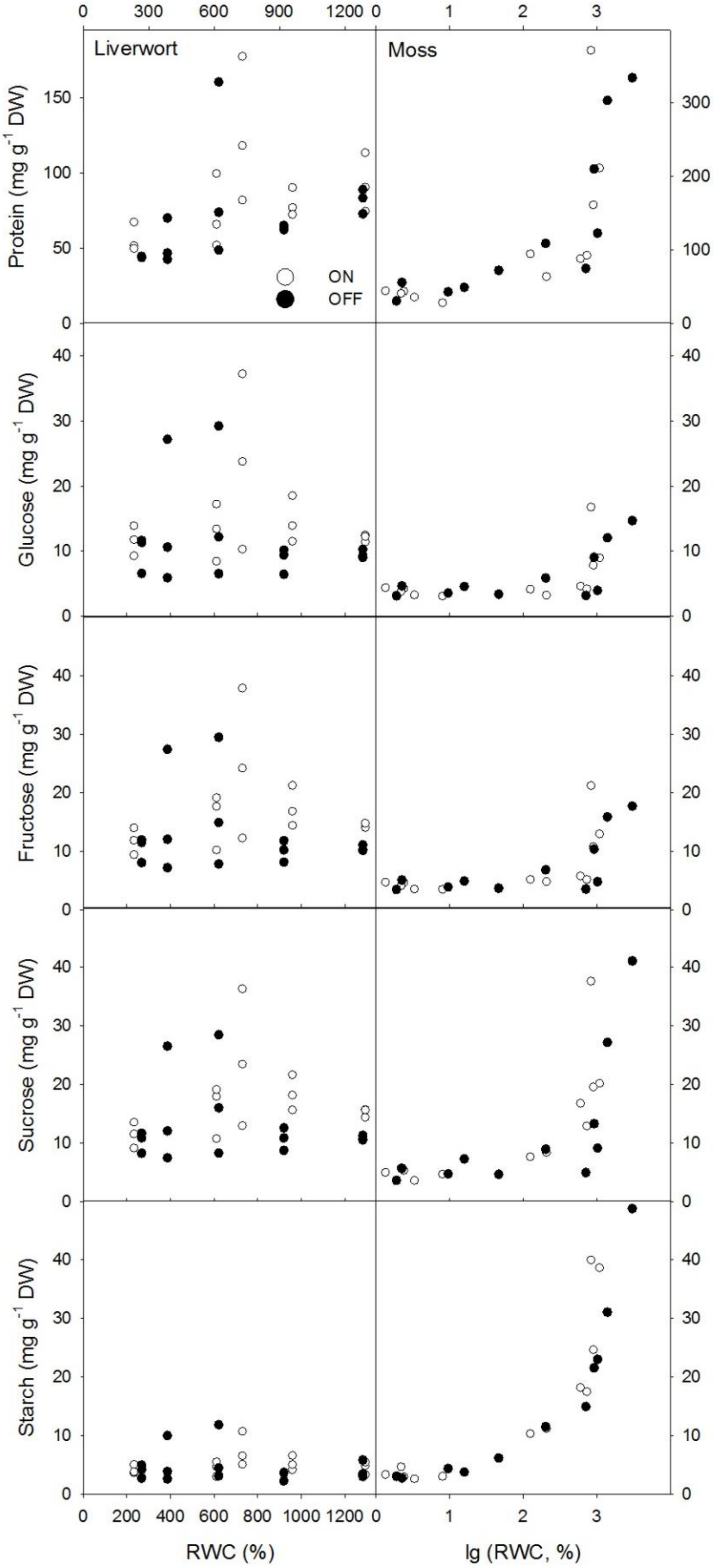

Liverwort
Moss
ON
OFF
Protein (mg g^{-1} DW)
Glucose (mg g^{-1} DW)
Fructose (mg g^{-1} DW)
Sucrose (mg g^{-1} DW)
Starch (mg g^{-1} DW)
RWC (%)
lg (RWC, %)

Fig. S5 CO_2 (A^C, A) and COS (A^S, B) net assimilation along decreasing tissue relative water content (RWC), in the dark, at two temperatures, in the liverwort (*Marchantia polymorpha*). Each symbol is an individual sample, the lines are smooth curves (fitted with a generalized additive model) and the shaded areas denote the 95% confidence interval for A^C and A^S at 16 °C (blue) and at 21 °C (red). Areas where the confidence interval do not overlap denote a significant effect at α = 0.05. Black areas denote the mean (± sd) fluxes of the blank chamber

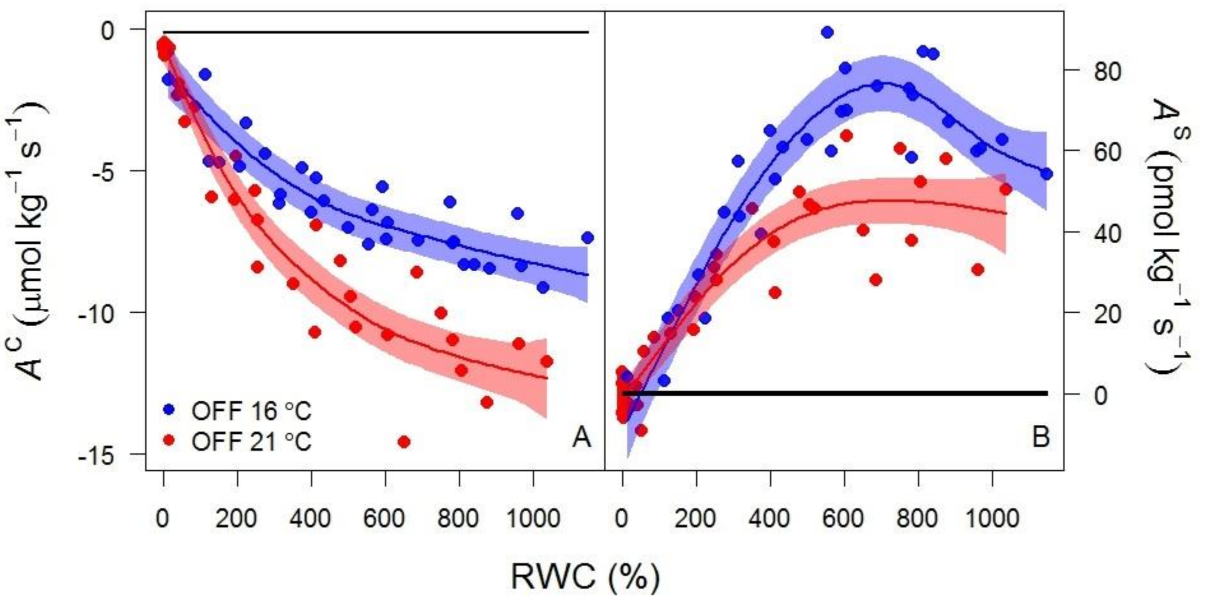

Table. S1 Estimated regression coefficients (with their 95% confidence intervals) and summary statistics (*t* and *P*) of the linear mixed models performed to assess the effects of COS mixing ratio (C^S in pmol mol^{-1}), in the liverwort (*Marchantia polymorpha*), in the light and in the dark ('Light'); and light intensity (PPFD in µmol m^{-2} s^{-1}) under ambient and near-zero C^S of the inlet air ('Source') on net measured CO_2 (A^C in µmol kg^{-1} s^{-1}) and COS assimilation (A^S), gross estimated COS uptake (U^S, the latter two in in pmol kg^{-1} s^{-1}). The response of A^C to PPFD was fitted to a non-linear function of the form: $A^C = R_d + A_{max}$ (1-$e^{-k\ \mathrm{PPFD}}$), where R_d is the estimated CO_2 emission in the dark, A_{max} is the maximum CO_2 uptake (both in µmol kg^{-1} s^{-1}) and *k* is a constant such that $A_{max}k$ is the slope of the light-limited part of the curve, a proxy for the light use efficiency

Variable	Factor	Estimate (95% CI)		*t*	*P*
		**Dark**	**Light**		
A^S	Light	-12 (-15, -9)	-21 (-26, -16)	-3.2	0.005
	C^S	0.059 (0.051, 0.068)		13.3	< 0.001
	Light x C^S	0.004 (-0.009, 0.018)		0.6	0.526
A^C	Light	-5 (-7, -3)	25 (19, 30)	11.1	<0.001
	C^S	-0.002 (-0.005, 0.001)		-1.4	0.182
	Light x C^S	0.001 (-0.004, 0.005)		0.3	0.748
		**Ambient**	**Zero COS**		
A^S	Source	120 (110, 131)	-4 (-6, -3)	-28.5	<0.001
	PPFD	-0.101 (-0.129, -0.067)	-0.024 (-0.029, -0.019)	-9.6	<0.001
	Source x PPFD	0.077 (0.049, 0.098)		6	<0.001
U^S	Source	130 (120, 141)	4 (2, 5)	-27	<0.001
	PPFD	-0.078 (-0.104, -0.042)	-0.012 (-0.016, -0.007)	-7.4	<0.001
	Source x PPFD	0.067 (0.037, 0.089)		5.2	<0.001
A^C	R_d	-11 (-15, -7)		-5.2	<0.001
	A_{max}	60 (55, 66)		22.4	<0.001
	k	0.008 (0.006, 0.01)		7.8	<0.001
	R_d		-10 (-17, -3)	-2.8	0.014
	A_{max}		50 (39, 61)	9.2	<0.001
	k		0.005 (0.002, 0.009)	3.2	0.005
